# Experiences and Factors Affecting Usage of an eHealth Tool for Self-Management Among People With Chronic Obstructive Pulmonary Disease: Qualitative Study

**DOI:** 10.2196/25672

**Published:** 2021-04-30

**Authors:** Sarah Marklund, Malin Tistad, Sara Lundell, Lina Östrand, Ann Sörlin, Carina Boström, Karin Wadell, Andre Nyberg

**Affiliations:** 1 Section of Physiotherapy Department of Community Medicine and Rehabilitation Umeå University Umeå Sweden; 2 School of Education, Health and Social Studies Dalarna University Falun Sweden; 3 Department of Neurobiology, Care Sciences and Society Karolinska Institutet Stockholm Sweden; 4 Karolinska University Hospital Solna Sweden

**Keywords:** COPD, qualitative content analysis, eHealth, self-management, primary care, chronic disease

## Abstract

**Background:**

Self-management strategies are regarded as highly prioritized in chronic obstructive pulmonary disease (COPD) treatment guidelines. However, individual and structural barriers lead to a staggering amount of people with COPD that are not offered support for such strategies, and new approaches are urgently needed to circumvent these barriers. A promising way of delivering health services such as support for self-management strategies is the use of eHealth tools. However, there is a lack of knowledge about the usage of, and factors affecting the use of, eHealth tools over time in people with COPD.

**Objective:**

This study aimed, among people with COPD, to explore and describe the experiences of an eHealth tool over time and factors that might affect usage.

**Methods:**

The eHealth tool included information on evidence-based self-management treatment for people with COPD, including texts, pictures, videos as well as interactive components such as a step registration function with automatized feedback. In addition to the latter, automated notifications of new content and pedometers were used as triggers to increase usage. After having access to the tool for 3 months, 16 individuals (12 women) with COPD were individually interviewed. At 12 months’ access to the tool, 7 (5 women) of the previous 16 individuals accepted a second individual interview. Data were analyzed using qualitative content analysis. User frequency was considered in the analysis, and participants were divided into users and nonusers/seldom users depending on the number of logins and minutes of usage per month.

**Results:**

Three main categories, namely, ambiguous impact, basic conditions for usage, and approaching capability emerged from the analysis, which, together with their subcategories, reflect the participants’ experiences of using the eHealth tool. Nonusers/seldom users (median 1.5 logins and 1.78 minutes spent on the site per month) reported low motivation, a higher need for technical support, a negative view about the disease and self-management, and had problematic health literacy as measured by the Communicative and Critical Health Literacy Scale (median [range] 154 [5-2102]). Users (median 10 logins and 43 minutes per month) felt comfortable with information technology (IT) tools, had a positive view on triggers, and had sufficient health literacy (median [range] 5 [5-1400]). Benefits including behavior changes were mainly expressed after 12 months had passed and mainly among users.

**Conclusions:**

Findings of this study indicate that the level of motivation, comfortability with IT tools, and the level of health literacy seem to affect usage of an eHealth tool over time. Besides, regarding behavioral changes, gaining benefits from the eHealth tool seems reserved for the users and specifically after 12 months, thus suggesting that eHealth tools can be suitable media for supporting COPD-specific self-management skills, although not for everyone or at all times. These novel findings are of importance when designing new eHealth tools as well as when deciding on whether or not an eHealth tool might be appropriate to use if the goal is to support self-management among people with COPD.

**Trial Registration:**

ClinicalTrials.gov NCT02696187; https://clinicaltrials.gov/ct2/show/NCT02696187

**International Registered Report Identifier (IRRID):**

RR2-10.1136/bmjopen-2017-016851

## Introduction

Chronic obstructive pulmonary disease (COPD) is a common, preventable, and treatable disease, listed as the third leading cause of death worldwide by the World Health Organization [1]. The disease most often presents with dyspnea of varying severity [2]. COPD is a complex disease with several pulmonary and extra-pulmonary manifestations, and leads to numerous negative clinical consequences such as exercise intolerance, decreased physical activity, decreased quality of life, as well as increased health care use [2,3].

Previous studies have shown that among people with COPD, those who use self-management strategies to manage their disease present with fewer symptoms and show reduced negative consequences of the disease [4-6]. Self-management strategies, such as exercise training, breathing strategies, and energy-conserving techniques during activities of daily life [7], have previously been found to reduce the need for hospitalization, increase physical activity and physical performance, as well as improve the quality of life [4-6,8,9].

Despite a high prioritization of self-management in COPD treatment guidelines [10], a staggering amount of people are not offered support for such strategies due to limitations of both individual and structural nature [10-12]. It is, therefore, crucial to find a way to circumvent this problem so that people with COPD are offered support to learn and use these COPD-specific self-management strategies.

eHealth tools, such as mobile apps and web-based platforms, represent a promising way of delivering health services such as support for self-management strategies to people with COPD. eHealth solutions are becoming increasingly common and are found to be feasible and effective within the COPD population [9,13-20]. eHealth tools have, for example, been used for educating and keeping track of a person’s health and are thought to be a significant source of health-related information [14,15,19,20]. We previously found that access to the COPD Web for 3 months resulted in increased self-reported levels of physical activity among people with COPD [17]. Improved COPD-specific knowledge and altered self-management strategies were also found among the participants. However, the general use of the COPD Web varied profoundly among participants, and the vast majority of users mainly used the platform during the initial month [17]. To date, several research groups have investigated and provided valuable information on important factors to consider when designing eHealth tools [21-23], though less is known about user behavior over time. Specifically, knowledge of factors associated with the use of eHealth tools over time is sparse. The aim of this study was, therefore, to explore and describe the experiences of using an eHealth tool over time and factors that might affect usage among people with COPD.

## Methods

### Study Design

This exploratory qualitative study is presented in line with the Consolidated Criteria for Reporting Qualitative Research (COREQ) guidelines [24]. This qualitative study is part of a process evaluation in a parallel-group (1:1 allocation) controlled pragmatic pilot trial that was reported in line with the Consolidated Standards of Reporting Trials (CONSORT) statements for pragmatic trials and for pilot and feasibility trials [25,26]. The study was registered at ClinicalTrials.gov (NCT02696187) and ethical approval was given by the Regional Ethical Board, Umeå University, Umeå, Sweden (Dnr: 2014-319-31, 2015-457-32). Written informed consents were obtained from each participant before enrollment in the study. This study includes participants that were allocated to the intervention group and that had access to an eHealth tool, the COPD Web. To further ensure privacy of participants, all names were changed to pseudonyms during the start of the analysis, so that only interviewers (AN and MT) and SM knew their real names.

### Setting and Sample

Five publicly funded primary health care units (2 situated in the north of Sweden and 3 in the middle of Sweden) were invited to participate as study sites in the pragmatic pilot trial [27]. In the main study 83 patients with COPD were included; of these, 43 were randomized to the intervention group and had access to the COPD Web. Of those randomized to the intervention group, a minimum of 3 participants at each of the 5 units were contacted and asked to participate in the individual interviews. We used purposeful sampling to ensure there is at least one male/female person with COPD at each primary health care unit. To be included in this trial, participants had to be able to read and understand Swedish or be assisted by someone with this capacity when using the eHealth tool.

### The COPD Web

In brief, the COPD Web is an interactive webpage cocreated with users [16]. It consists of 2 main sections: one directed at health professionals, and another directed at people with COPD. Contents include videos, written information, images, and helpful links. The COPD Web section aimed at people with COPD to support their self-management by increasing their knowledge about COPD and strategies to improve their health (eg, activities such as physical activity and exercise, breathing techniques, observing symptoms of exacerbations, and advice about making everyday activities less strenuous) [27]. In addition to the specific content of the COPD Web, there were a few things on the fringe of what was covered by the COPD Web that should be noted. The COPD Web also includes a function of registering physical activity (steps), for which participants received a pedometer with instructions on how to use it as well as an information leaflet on the importance of physical activity [27]. The COPD Web also had automated notifications of new publications on the website via email. It was first introduced to each participant by a health professional during a regular visit using a standardized procedure (designed to take a maximum of 10-15 minutes). During the introduction of the COPD Web, all participants were provided with a username and login. Further information about the eHealth tool is presented in the protocol [27].

### Process of Data Generation

Overall, 16 participants (12 women), age range 48-86, accepted to participate in an individual face-to-face interview at 3 months after the intervention started. Follow-up interviews were done at 3 and 12 months, as both ranges of time are commonly used when investigating intervention-based effects in people with COPD [13,14]. At 3 months, 15 interviews were conducted in the participants’ homes and 1 at a university (the worksite of the interviewer), according to the participant’s wishes. Sociodemographic information including age, sex, civil status, occupation, physical activity, smoking habits, and educational level was obtained through a standardized questionnaire while information on lung function was obtained from medical records [27]. At the 12-month follow-up, the participants were contacted again over the telephone and 7 (5 women) of the previous 16 participants accepted a second individual interview—this time conducted over the telephone. The reason for declining a second interview was that they had not used the COPD Web at all between the 3- and 12-month follow-up period.

Experiences related to the usage of the COPD Web over time were collected through individual semistructured qualitative interviews. An interview guide was used as the framework for the interviews. It consisted of the following main areas: (1) user habits, (2) user experience, (3) accompanying parts (ie, introduction, pedometer, electronic newsletter), (4) potential effects, and (5) future use (Multimedia Appendix 1). Questions regarding all main areas were posed, albeit in varying order. The interviews at the 3-month follow-up ranged between 7 and 40 minutes (median 25 minutes) and interviews at 12 months ranged between 4 and 14 minutes (median 8 minutes).

In addition, data on characteristics of the individuals and thought to be important for their use of the COPD Web were collected. It included the impact of COPD on daily life measured with the COPD Assessment Test [28], dyspnea measured with the modified Medical Research Council Scale [29], and health literacy (ie, the degree to which individuals can find, understand, and use information and services to inform health-related decisions and actions for themselves and others) [30]. The latter was measured with the Communicative and Critical Health Literacy (CCHL) Scale questionnaire. A total score of less than 100 indicates a sufficient communicative and critical health literacy [30], a total score of more than 100 but less than 1000 indicates a problematic health literacy, and a total score of over 1000 indicates a lack in communicative and critical health literacy [30]. Data on the participants’ use of the COPD Web were collected automatically from the website and included the number of visits (logins), pages used, and time spent on the website [27].

### Research Team and Reflexivity

AN (PhD in physiotherapy, male, 32 years) and MT (PhD in physiotherapy, female, 43 years), conducted the interviews separately depending on geographic placement. Both interviewers were employed as postdoctoral researchers at Umeå University at the time. Prior to the study, AN had performed over 20 interviews (no specific training), and MT had performed over 30 interviews (supervision during postdoctoral employment). There was no relationship established between the researcher and the participant before the interviews.

In all of the interviews, only the researcher and the participant were present, and audio recordings were used. MT made short field notes for most of the interviews (used as a mean of recollection during analysis), AN did not take notes during or after the interviews. No immediate callbacks on the interviews were made (ie, for potential amendments or additional questions). Interesting or unexpected topics raised during the interviews were discussed and used to guide follow-up questions during the following interviews. Interview audio recordings were transcribed verbatim by an experienced third-party transcriber and verified by the authors by comparing with the audio records [31]. Transcripts were not returned to participants for comment or correction. Participants were not engaged to provide any feedback on the findings.

### Analysis

Data were analyzed using an inductive approach of qualitative content analysis, as this method is useful when dealing with already gathered qualitative data where the goal is to increase the understanding of experiences of using an eHealth tool [32]. All interviews were read through several times (with the assistance of audio recordings for auditory cues). The interviews were then condensed, coded, and sorted into categories and subcategories [33]. MAXQDA 2018 software was used in the analysis process to facilitate administration of the interviews, codes, and quotes. SM was main responsible for the analysis, and researcher triangulation was used throughout the whole analysis phase to attain a higher level of credibility [16,33]. Continuous discussions among authors during the interviewing phase and making use of a semistructured interview guide were measures taken to enhance dependability [33].

To further explore usage over time, the participants were subgrouped based on their objective use of the COPD Web during the initial 3 months. *Users* were defined as those with more than 1 login/month or more than 20 minutes total spent time/month at 3 months. An individual that did not meet these criteria were defined as a *nonuser/seldom user* at 3 months. Codes by users and nonusers/seldom users were then marked in the subcategories to analyze the data further. Furthermore, to explore usage during the initial 3-month period, we also compared responses between the 3- and 12-month interview in those accepting a follow-up interview at 12 months.

## Results

### Study Participants

Patients with COPD at each of the 5 included primary health care units were contacted with the goal of recruiting at least one female/male person with COPD at each unit. No male participant accepted an interview at one of the primary health care units. In total, we contacted 23 patients with COPD (12 female); of these, 7 declined (2 female), and 16 accepted participation in the interviews at 3 months. Furthermore, of those who accepted an interview at 12 months, 5 out of 7 were users at 3 months. When subgrouped based on their use of the COPD Web during the initial 3 months, users had, in median, sufficient communicative and critical health literacy. By contrast, nonusers/seldom users had problematic communicative and critical health literacy. No other apparent differences in absolute values were seen for any other participant characteristics between users and nonusers/seldom users at 3 months (Table 1).

**Table 1:** Participant characteristics.

Characteristics		Interview at 3 months (n=16)	User at 3 months (n=6)	Nonuser/seldom user at 3 months (n=10)
Age (years), median (range)		71 (48-86)	70 (48-86)	72 (54-81)
Sex (female), n		12	5	7
FVC^a^ predicted (%), median (range)		80 (59-131)	83 (71-131)	71 (59-126)
FEV_1_^b^ predicted (%), median (range)		61 (30-99)	60 (54-99)	61 (30-93)
FEV_1_/FVC (%), median (range)		52 (28-63)	56 (47-63)	46 (28-62)
CAT^c^, median (range)		13 (2-20)	15 (2-17)	12 (2-20)
MRC^d^, median (range)		1 (0-4)	1 (1-3)	1.5 (0-4)
**Stage of COPD^e^, n (%)**				
	A	3 (19)	1 (17)	3 (30)
	B	7 (44)	4 (67)	3 (30)
	C	2 (13)	1 (17)	1 (10)
	D	3 (19)	0 (0)	3 (30)
**Smoking status^f^**				
	Never smoker, n (%)	0 (0)	0 (0)	0 (0)
	Ex-smoker, n (%)	13 (87)	6 (100)	7 (78)
	Current smoker, n (%)	2 (13)	0 (0)	2 (22)
	Pack-years, median (range)	24 (6-55)	18 (6-20)	29 (15-55)
**Employment status^f^, n (%)**				
	Currently working	3 (20)	2 (33)	1 (11)
	Retired	12 (80)	4 (67)	8 (89)
	Sickness benefits	0 (0)	0 (0)	0 (0)
**Living with^f^**				
	Alone, n (%)	11 (73)	5 (83)	6 (67)
	Family, n (%)	4 (27)	1 (17)	3 (33)
**Education level^f^, n (%)**				
	Primary	9 (60)	4 (67)	5 (56)
	Secondary	3 (20)	1 (16.5)	2 (22)
	Tertiary	3 (20)	1 (16.5)	2 (22)
**Health literacy test**				
	CCHL^g^ scale (score), median (range)	104 (5-2102)	5 (5-1400)	154 (5-2102)
**Usage**				
	Minutes/month, median (range)	7.8 (0-144.6)	42.7 (26.8-144.6)	1.8 (0-16.4)
	Logins, median (range)	4 (0-33)	10 (5-33)	1.5 (0-14)

^a^FVC: forced vital capacity.

^b^FEV_1_: forced expiratory volume in 1 second.

^c^CAT: COPD assessment test (0-40, higher scores denote a more severe impact of COPD on patient’s daily life).

^d^MRC: Medical Research Council Scale (1-5, higher score denote a higher degree of disability that breathlessness poses on day-to-day activities).

^e^COPD: chronic obstructive pulmonary disease.

^f^Data missing on 1 participant.

^g^CCHL: Communicative and Critical Health Literacy Scale, Swedish version (lower score = better health literacy).

**Figure 1:**
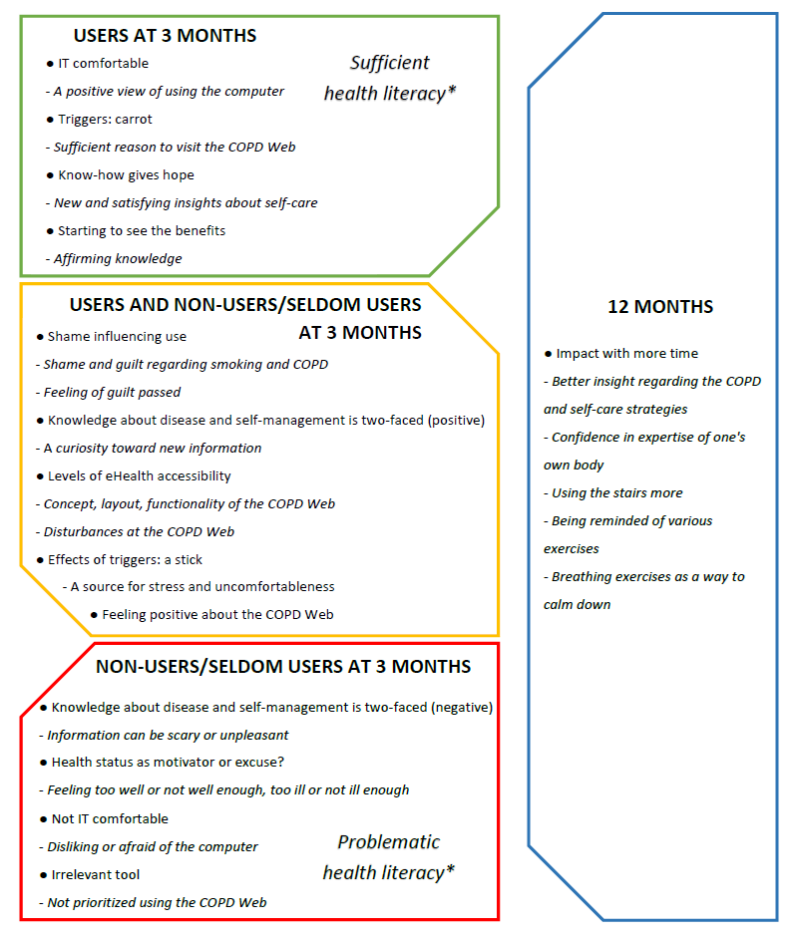
Overview of user experience and factors affecting usage. “Users at three months” represent experiences mainly seen among those who used the COPD Web during the initial three months, “Non-user/seldom-users at three months” represent experiences mainly seen among those who did not use, or used at a limited extent, the COPD Web at three months, “Users and non-user/seldom-users at three months” represents experiences seen among both users and non-user/seldom-users at three months while “12 months” represents experiences mainly expressed at 12 months. *Based on the communicative and critical health literacy scale, Swedish version.

The analysis resulted in 3 main categories, which, together with the subcategories, represent the participants’ overall experiences regarding the use of the COPD Web over time and descriptions of factors that could affect the usage of the said tool (Textbox 1). An overview of the main results are seen in Figure 1.

**Description of categories and subcategories. IT: information technology.**

**Ambiguous impact**

- Shame influencing use
- Knowledge about disease and self-management is two-faced
- Health status as motivator or excuse?
- IT comfortable or not?

**Basic conditions for usage**

- Levels of eHealth accessibility
- Triggers: carrot or stick?
- Feeling positive about the COPD Web
- Irrelevant tool

**Approaching capability**

- Know-how gives hope
- Starting to see the benefits
- Impact with more time

### Ambiguous Impact

#### Overview and Subcategories

In this category, participants expressed contradicting thoughts about seeking care due to, and specific information about, COPD. There were also some diverging thoughts on competence and motivation regarding the use of information technology (IT). The category includes the 4 subcategories “Shame influencing use,” “Knowledge about disease and self-management is two-faced,” “Health status as motivator or excuse?,” and “IT comfortable or not.”

#### Shame Influencing Use

Having COPD was sometimes experienced as something taboo, hushed up, or embarrassing which led to loneliness or isolation and prompted nonusers/seldom users to distance themselves from the eHealth tool, and users to try out the tool. A sense of not having anyone to talk to about their COPD, and feeling like COPD is rarely talked about, were mentioned as reasons for liking and welcoming the COPD Web.

Yes, but just talking about COPD, well...you just actually don’t do that. That’s why it’s a good thing with this kind of page where you can get to know a little more.

Charlotte, user, 3-month interview

Among nonusers/seldom users experiences of shame and guilt regarding smoking and COPD were conveyed. A feeling of shame caused participants to withhold the diagnosis from family and friends and also hindered contacting health care. Having developed COPD was described as one’s own fault (due to smoking), and therefore, something one should handle all by oneself. Hence using the COPD Web might be seen as getting undeserved assistance.

Well, this...I only have myself to blame. This is a result of my smoking. And that’s how it is. I guess I’ll have to battle this on my own.

Gertrud, nonuser/seldom user, 3-month interview

#### Knowledge About Disease and Self-Management Is Two Faced

Participants proclaimed a curiosity toward new information, a wish to know more, and indeed reported having actively searched for information and sources of information. Only nonusers/seldom users expressed that the urge to receive information about COPD and COPD-related subjects was not absolute. In addition, users mentioned the advantage of having access to a lot of information, whereas nonusers/seldom users felt that information could be scary or unpleasant, especially if it contradicted one’s preconceptions. At the 12-month interview, participants (both users and nonusers/seldom users at 3 months) mentioned a desire to see if any new information had been posted or to refresh knowledge on something they had read about earlier as reasons for revisiting the COPD Web.

#### Health Status as Motivator or Excuse?

The own health status experienced was a prominent reason to visit or not to visit the COPD Web (ie, feeling good or poor made them more likely to use [or not use] the COPD Web). One view was that if one felt too well he/she would not be motivated to search for information, while another view was that one had to feel good to really be able to take in the advice and information. Besides, some thought themselves not ill enough while some proclaimed themselves not ill at all—either way, none of these conditions seemed to warrant visits to the COPD Web.

Well, of course, the better you feel…I believe it’s more like you think it’s more fun to just go in and read.

Bella, nonuser/seldom user, 3-month interview

[…] I kind of feel too healthy.

Katarina, nonuser/seldom user, 3-month interview

At 12 months it was still thought by former users to be more likely that one will use the eHealth tool if he/she feels sick.

#### IT Comfortable or Not?

Participants had different comfort levels regarding the use of computers and the internet. A positive view of using the computer and the internet was expressed among users. Users further mentioned the advantage of the COPD Web being accessible from anywhere (at home and any other place on the globe). Disliking or being unused to the computer or the internet was only mentioned by nonusers/seldom users as hinders for visiting the COPD Web and searching for information in general. Later, at 12 months, a nonuser/seldom user that previously stated her dislike and ignorance regarding both computers and the internet said that she was somewhat afraid of the computer:

Actually, I’m also a little afraid of the computer […] l am reluctant to have anything to do with it. Unfortunately. Sometimes I think that now I will…and then… “No, not now, not now, not now!”

Penny, nonuser/seldom user at 3 months, 12-month interview

Nonusers/seldom users also reported not prioritizing using the computer or the COPD Web, not having any interest in computers or the COPD Web, and preferring to read information as well as note (eg, step counts) on a paper instead of on the computer screen. Others explained their absence from the website as a result of being engaged in other activities or having forgotten about the computer or the COPD Web. Still, comments on lack of time for using the computer or COPD Web occurred among nonusers/seldom users. Disinterest for the eHealth tool was also reported at 12 months by both users and nonusers/seldom users.

Nonusers/seldom users expressed a need for their support system in the form of health professionals or close relatives. Support was used as a source of knowledge when it came to using the technology involved, as well as a motivational source for looking up information or learning new things and for allocating time for computer use.

I’m lucky to have a couple of lads I can call. “What the hell do I do now?”

Kenneth, nonuser/seldom user, 3-month interview

### Basic Conditions for Usage

#### Overview and Subcategories

This category represents the technical aspects of the eHealth tool, covering both experiences of the functionality of the software and hardware and various attitudes using technical products in general. This category includes the 4 subcategories “Levels of eHealth accessibility,” “Effects of triggers: a carrot or a stick?,” “Feeling positive about the COPD Web,” and “Irrelevant tool.”

#### Levels of eHealth Accessibility

Generally, the concept, layout, and functionality of the COPD Web was appreciated at 3 as well as 12 months. Participants described disturbances, such as problems with their computer, frustrating and inconvenient. Sometimes strong feelings of being fed up with things not working were expressed, at both 3 and 12 months.

No, there’s nothing else. There’s my computer. I would be out on the web almost every other day looking, otherwise.

Carol, nonuser/seldom user, 3-month interview

Well, I’ve had a few problems with my computer, but I have been in and checked all [e-mail newsletters] that I’ve received.

Cindy, user at 3 months, 12-month interview

The COPD Web’s low compatibility with smartphones was reported as a hindrance by some nonusers/seldom users at both 3 and 12 months.

#### Triggers: Carrot or Stick?

Overall, participants felt that the triggers (ie, automated notifications of new content, pedometer, and the step registration function) had a kind of gateway function and made using the COPD Web closer at hand. Users described themselves positively about using and subsequently revisiting the COPD Web when getting email newsletters from the eHealth tool.

The pedometer was reported to be a useful tool concerning physical activity; it was also perceived as a sufficient reason to visit the COPD Web to register steps on the site. Using the pedometer was recognized as a way to prove, in a concrete way, that you had done right (or done something at all) with regard to your level of physical activity. There was a drive, a motivation, expressed in registering steps on the COPD Web, and a pedometer helped in building this motivation.

My [pedometer] was my dangling carrot. All the time, I could see how much I walked … well, I thought that was really terrific.

Patricia, user at 3 months, 12-month interview

However, the pedometer was also recognized as a possible source for stress and uncomfortableness if you had not “done enough.” Some nonusers/seldom users indeed felt like being monitored or watched when wearing it, which could be experienced as both a motivational push and something negative. Further, the pedometer was thought by some users to be of more use to someone worse off, health-wise, than oneself.

Overall, participants expressed that the introduction to the COPD Web had included a suitable type and amount of information and also that the health professionals had introduced the COPD Web well, both elements triggering interest for the COPD Web. Among nonusers/seldom users, getting valuable support during the introduction which inclined them to participate despite feeling insecure about computer use was expressed. Further, some users reported having participated due to a sense of duty. However, a somewhat contradictory experience was also conveyed, expressing that the introduction took too much time and was not relevant:

Because I’m not ill. So I shouldn’t have to be there (at the introduction). And so I thought … good God, does this [introduction] never end?

Gertrud, nonuser/seldom user, 3-month interview

#### Feeling Positive About the COPD Web

Users of the COPD Web expressed that they were generally missing feedback from the health care provider/system regarding the actions of self-care they had already set in motion and were glad to be able to verify these with the help of the COPD Web. Possible prevention of deterioration was expressed as reason enough for using the COPD Web for some users. Users also mentioned that one can choose what to focus on, or do, on the COPD Web, and this was remarked as a very positive feature/function of the site. Further, users of the website reported liking how it can remind you about information, exercises, and other supportive tools for self-management. The COPD Web was also regarded as a source for positive extrinsic motivation by both users and nonusers/seldom users. Participants expressed curiosity for the COPD Web, as well as positive anticipatory feelings toward it. The COPD Web was described as a new concept that had not been introduced to them before and which spiked their interest.

[…] I thought … my God, that’s great! This is about me. Naturally I needed to grip the chance. That’s how it was. And the way in which [the COPD nurse] presented things and the way [the COPD nurse] described things caught my interest […]

Kelly, user, 3-month interview

While the thought of appreciating all help that you can get was mentioned, it did not ensure the usage of this eHealth tool. Participants mentioned that looking around in general and checking what the site was about as reasons for visiting the COPD Web.

#### Irrelevant Tool

Nonusers/seldom users felt that they did not have time for, had forgotten about, or had otherwise not prioritized using the COPD Web. Some users also expressed this feeling. A sense of having been “in the game” for too long, and thus already having heard all the information was expressed. This was also mentioned at 12 months. Some felt this was fine, and some were frustrated about not being able to find new information, but this ultimately seemed like a reason not to “bother” with the eHealth tool. Others felt that they were already doing what the COPD Web instructed them to do regarding self-care and so had less need for the website. Consequently, the website was expected to feel more relevant when you are newly diagnosed. In addition, visits on the website could be due to a sense of obligation (toward the study) as this former user stated at 12 months when asked how come they stopped their, formerly very active, step registration:

Yes, but then I asked you whether […]… should I continue registering my steps? You said, no, you do as you want to. […] So then I didn’t bother with it.

Cindy, user at 3 months, 12-month interview

### Approaching Capability

#### Category Overview

This category includes participants’ experiences regarding getting access to COPD-specific knowledge and skills for self-management and the benefits experienced from using the COPD Web, as well as reports of actual behavioral changes within 12 months since starting to use the COPD Web. The 3 subcategories covered are “Know-how gives hope,” “Starting to see the benefits,” and “Impact with more time,” wherein the for the last subcategory all codes are solely from the later (12-month) follow-up.

#### Know-How Gives Hope

The content of the COPD Web was perceived to be instructive in a concrete way, which gave new and satisfying knowledge about and insights into self-care. Users reported using the website as a sort of archive where they could search for information and tips, even regarding things that were not specific to COPD. Some users felt it was nice just to have access to this well of information, seemingly regardless of how much it was utilized. A remark on the power of habit was made where the use of the COPD Web felt helpful in changing or affecting habits in a good way because it could be revisited several times. Users also perceived that their view of COPD had become more hopeful because the COPD Web had informed them about the things that they were able to use to their advantage. Having heard depressing statements from people in one’s vicinity, it felt positive to learn about actions to take and feelings of being fortified and boosted in your actions of self-care.

Well, do you want to know what is good? That there is … that you don’t have to become worse and worse but there is something you can do about it yourself. I think that’s positive

Cindy, user, 3-month interview

By contrast, some users conveyed a sense of no change at all in their view of the disease from having access to the COPD Web.

#### Starting to See the Benefits

To get an affirmation of what you had already heard or learned from other sources was an appreciated function of the COPD Web, and this is in line with the statement of some users that not all advice on the website is new news, but they can still help you. Otherwise, users felt they visited the website to improve their overall situation by learning more about the diagnosis, their body and treatments, and how to perform a specific exercise. Breathing techniques on the site were reported as helpful during, for example, travels and also as a helpful way to prepare before a particular trip.

I looked at this thing about going up steps. And so I went to Spain and I knew that we had to go up some steps a long way up to a church and so I actually practiced exhaling on the next step. You breathe in, take a step and then breathe out and take two steps and so on. I think this worked very well.

Patricia, user, 3-month interview

In addition, the COPD Web function of step registration was mentioned as a strong motivator for doing exercises.

#### Impact With More Time

In this subcategory, a little more time had passed, and all statements are from the 12-month follow-up. The content of the website was said to have led to a better insight regarding the COPD and the self-care strategies, as well as to essential changes in everyday life. Participants commented that the COPD Web had helped them in understanding how vital actions such as contacting the health care in time and using the stairs were important. However, learning about COPD, in general, had also given some confidence in the expertise one holds about one’s own body. Therefore, it was said, one could also feel more confident in asking for, or even demanding, care from their health care settings when something did not seem right.

Now, just like then when I was so wilted at Christmas time there, I was the one who stood up for myself and told them that this is the way I am and I now need to come in and perhaps get some help to stop all this. Yes. Not to say… and other times I’ve let things drag on more. That’s just the way it is. But I do understand that it’s more important to [seek care] quicker and not let things drag on.

Kelly, user at 3 months, 12-month interview

Others said that changes to everyday life were the actively changed patterns of body motions (eg, standing up without using one’s hands), using the stairs more, and avoiding getting minor illnesses that might escalate to worse conditions due to COPD. Some felt that they had learned more from the website than from traditional health care meetings. The convenience of being reminded of various exercises and breathing exercises was especially mentioned as a way to calm down, to feel more at ease. By contrast, there were participants, including both former users and nonusers/seldom users, who after the 12 months still felt that their habits had not changed and that things were as they always had been—with or without the COPD Web.

## Discussion

### Principal Findings

This study aimed to, among people with COPD, explore and describe the experiences of using an eHealth tool over time and factors that might affect usage. The main results reflected study participants’ experiences in the categories of *ambiguous impact*, *basic conditions for usage*, and *approaching capability,* and indicated that level of motivation, comfortability with IT tools, and the level of health literacy seem to affect usage of an eHealth tool over time. Furthermore, regarding behavioral changes**,** gaining benefits from the eHealth tool seems reserved for the users and specifically after 12 months, thus suggesting that eHealth tools can be suitable media for supporting COPD-specific self-management skills, although not for everyone or at all times. These findings support earlier qualitative studies noting that people with COPD are a heterogeneous group and that there is no one general way to reach all people with COPD [34]. Furthermore, even though technical difficulties are irritating, they do not seem to determine level of usage. Similarly, recognizing the usefulness of the eHealth tool, and even curiosity toward the tool do not seem to influence the level of usage.

### Interpretation of Findings: User Experience and Factors Affecting Usage

In general, users shared positive comments about IT tools on the COPD Web, including receiving information, the electronic newsletters, and the contents on the eHealth tool. These findings are in line with previous studies in which those who used a mobile app, in general, had a positive view on eHealth tools [35,36]. Furthermore, as captured in the subcategory “Starting to see the benefits,” users seemed to be able to incorporate the advice into their everyday life, and they felt that the eHealth tool helped them to improve their overall situation. This suggests that the eHealth tool provided participants with at least some of the tools needed to manage their condition [7].

As knowledge and skill on how to integrate the demands of the disease into the daily routine are crucial to enable behavior modification [7,37], findings from this study are of importance. Specifically, in a similar way, as previously reported after self-management interventions for people with COPD, users in our trial reported increased knowledge on how to perform specific exercises and use of breathing techniques in daily life [38]. Some users also mentioned that they gained a sense of hope, as the eHealth tool had informed them about their disease so that they were able to utilize this knowledge to their advantage. The latter is of importance as a person’s belief in his or her ability to manage the disease is a powerful and well-recognized predictor of health-related behavior changes [7,39]. Our findings also suggest that the eHealth tool was beneficial as a self-management tool for the users, as it seemed to have resulted in more substantial confidence in their ability to manage their disease, which is an essential factor to influence specific health behaviors [40-43]. The sense of hope in itself could also be a reason for the continued use of the eHealth tool among users because the perception of a clear benefit has been highlighted as a critical facilitator for eHealth usage.

In addition to the positive view on information and IT tools, when exploring potential differences in baseline characteristics among users and nonusers/seldom users, we saw that users also had sufficient communicative and critical health literacy as measured by the CCHL Scale, Swedish version. Health literacy has been defined as “the degree to which individuals can obtain, process, and understand basic health information and services needed to make appropriate health decisions.” [44]. It is, in other words, a set of fundamental skills for obtaining precisely the kind of information that is communicated with the help of eHealth tools, which might help explain some basic differences between those who used and did not use the COPD Web. By contrast, nonusers/seldom users of the eHealth tool had a problematic communicative and critical health literacy. Thus, the level of health literacy might be a contributing factor to our observed findings because this is important in taking in new information as well as highlighting a need for education and training. The latter has also been identified as a critical barrier to eHealth use in daily practice [30]. Limited health literacy toward eHealth was likewise the top mentioned barrier for implementing eHealth services in a recent systematic review and meta-analysis [36]. Low health literacy is also widespread among patients with COPD. For example, Puente-Maestu et al [45] found that more than 50% of their patients with COPD had low health literacy. Similarly, as evident among nonusers/seldom users in our trial, people with both COPD and low health literacy have previously also been reported to be more dependent on others and to be more concerned about their illness [45,46]. With this in mind, our findings further support previous views that health literacy should be considered when designing self-management support programs for people with COPD [47] and, specifically, that low health literacy seems to be an important barrier to using eHealth tools over time and other strategies should be considered. Moreover, participants expressed that the COPD Web would have been especially relevant when they were newly diagnosed and that the COPD Web would have enabled them to circumvent underestimating the importance of self-care–related information. This observation is similar to the findings made by Ansari et al [48] who demonstrated that newly diagnosed patients with COPD had difficulty recognizing the impact of COPD on their health, mostly due to low awareness of the disease and its long-term implications.

Furthermore, nonusers/seldom users reported a problematic attitude toward IT tools or a completely nonfunctioning computer or internet connection at their disposal. However, even though nonusers/seldom users had access to and utilized help when needed, they still did not use the eHealth tool, which implies that their lower level of interest (low motivation) or faith in new information and technology had more to do with not using the tool more. Triggers, such as the automatic newsletters or pedometer, did not seem to affect usage in itself. The electronic newsletters, for example, did help in getting people to use the eHealth tool actively—but still, it only seemed to make a positive impact for those already more inclined to receive new information, use the computer/internet, and prioritizing an eHealth tool such as this. The same goes for the pedometer and the step registration function that were expressed as motivating and useful by users. These findings could be related to the Fogg Behavior Model (FBM) [37]. The FBM suggests that acquiring a targeted behavior, in our case, using the eHealth tool, requires sufficient motivation, ability, and triggers. Thus, the low motivation, the low ability to use IT tools, and the opposing view on used triggers help us understand why nonusers/seldom users did not use the tool.

Nevertheless, as reported above and captured in the subcategory “IT comfortable or not,” the expressed need for assistance could also be linked to the low health literacy among our nonusers/seldom users [45,46,49]. Further, no apparent differences in disease severity or other sociodemographic characteristics such as age, sex, smoking history, living conditions, or basic educational level could be seen between users and nonusers/seldom users of the eHealth tool. This further indicates that our population either used their health status as an excuse for not using the eHealth tool or that their perceptions of their health status varied no matter the objective measurements. Our findings, however, still indicate that the nonusers/seldom users were in that group more due to insufficient self-competence and motivation, and a more negative general attitude toward getting informed. For example, in contrast to users that only expressed a positive attitude toward receiving new information, nonusers/seldom users also expressed that information could be scary or unpleasant. Again, these findings could be linked to the lower health literacy of nonusers/seldom users [45,46,49]. In this study, both users and nonusers/seldom users expressed that the eHealth tool was easy to use. This indicates that even though the ease of use has been stated as the most successful factor for implementing eHealth services [36], it was not a key factor explaining usage in our sample.

In the interviews performed at 12 months, we could see a shift in the type of information provided in the interviews, despite using the same interview guide as during the 3-month interviews. Specifically, as highlighted in the category “Assembling know-how,” the reported changes in behavior (eg, standing up without using one’s hands and using the stairs more) were mainly expressed at the 12-month interviews. Thus, knowledge from the eHealth tool was put into practice, and behavioral changes were underway at this time. Even though participants, mainly users, also expressed benefits since having access to the eHealth tool after the initial 3 months, these findings highlight that time might be an essential factor when evaluating the potential benefit of eHealth tools. These findings are in line with the results from a recent previous study suggesting that a continuous self-management program helps people with COPD to perform given self-health behaviors, thus indicating that achieving changes in health behavior takes time [38]. Further support for the latter is also seen in a qualitative systematic review from 2018 [34]. Other than this observation, no apparent differences were seen between the 3- and 12-month interviews, which also supports the consistency of our 3-month findings [31,50].

### Strengths and Limitations

Strengths of this study are the direct comparison of users and nonusers/seldom users, as well as the design of the study following the COREQ guidelines, increasing the credibility of our findings [24]. Trustworthiness has been strived for by utilizing triangulation and recurrent discussions regarding the material through all phases [51]. Furthermore, interviews were conducted via both face-to-face meeting and telephone due to practical choices. The 2 interview methods are, however, equally credible according to Ward et al [52] who found that interviewees felt free to divulge sensitive information over the telephone and that the communicative cues were to be regarded as equal to those used within face-to-face interviews. Interview lengths are varying and at times, very short. However, a specific duration is not a guarantee for richness [51,53], and we interpreted our data to be rich enough for the analysis that was carried out. To counteract the potential loss of information by removing the researchers from this process by using a professional transcriber [54], the transcripts were then checked for deviations between audio and transcript [31]. Besides, knowledge and notes from the interviewers were utilized when needed during analysis. In addition, we continuously consulted the audio recordings when triangulation indicated risks of interpretational differences of a transcript [33]. Even though the number of interviews in itself is not a crucial criterion in qualitative methods, it should be noted that the number of informants, especially at 12-month interview, was relatively few. Besides, even though there was a variation in age, sex, education, stage of COPD, and living conditions, the vast majority of included participants were females, living alone, and with overall low disease severity as evident by low CAT and MRC scores, as well as moderate forced expiratory volume in 1 second (%FEV_1_) of predicted values. It should also be noted that the majority of the interviewees were women, highlighting a potential selection bias [55], as more men than women declined to participate in the interviews. Analyzing whether the results would have been similar if more men had participated was outside the scope of the study, but should be considered for future projects. In addition, of importance, the cut-offs used to define users and nonusers/seldom users were not predetermined and set by the researchers after having access to information about the usage of the COPD Web. However, these cut-offs were set by a researcher not involved in the primary analysis, and before codes were tracked back to individual informants. Lastly, objective data on the use of the eHealth tool were only available during the initial 3 months. Thus, we cannot elaborate on whether the users at 3 months also were users at 12 months.

### Conclusions

To our knowledge, this study was among the first to explore experiences and factors that affect the usage of an eHealth tool aiming at improved self-management strategies in people with COPD over time. The novel findings of this study indicate that usage of an eHealth tool, to support self-management, is affected by the view on the information. That is, curiosity or fear for new information can influence whether to use an eHealth tool or not. In addition, a low motivation, a higher need for technical support, and a problematic health literacy were seen among nonusers/seldom users. By contrast, users were comfortable with IT tools, had a positive view on triggers, and had sufficient health literacy. Lastly, reaping benefits from an eHealth tool seems to be reserved for the users, and primarily after 12 months. These findings suggest that eHealth tools can be suitable media for supporting COPD-specific self-management skills, although they are not for everyone or at all times.

Furthermore, factors such as motivation, IT comfortability, and level of health literacy are important to consider when deciding on whether or not an eHealth tool might be appropriate to use if the goal is to support self-management among individuals with COPD. In addition, our findings highlight that time might be an essential factor when evaluating the potential benefit of eHealth tools aiming at behavioral changes regarding self-management strategies.

## Appendix

Multimedia Appendix 1 Interview guide for people with COPD that have used the COPD Web.

## Abbreviations

CAT: COPD Assessment Test
CCHL: Communicative and Critical Health Literacy Scale
COPD: chronic obstructive pulmonary disease
COREQ: Consolidated Criteria for Reporting Qualitative Research
FBM: Fogg Behavioral Model
FEV: forced expiratory volume
FVC: forced vital capacity
MRC: Medical Research Council
IT: information technology
