# Supplementary material for: Experiences and Factors Affecting Usage of an eHealth Tool for Self-Management Among People With Chronic Obstructive Pulmonary Disease: Qualitative Study
Source: J Med Internet Res. 2021 Apr 30;23(4):e25672. doi: 10.2196/25672 (PMC8122287; doi:10.2196/25672)
Supplement: Multimedia Appendix 1 [file jmir_v23i4e25672_app1.pdf]

Appendix A

Interview guide for people with COPD that have used the COPD Web

Interview parts	Examples of questions
User habits (with starting question)	Tell me about how you have used the COPD Web (during this time)<br>In what purpose/purposes have you visited the COPD Web?
User experience	What are your views on having the computer/this kind of computer based program as help/assistance?<br>Have you missed anything particular on the COPD Web?<br>Have you been able to identify yourself with the contents on the COPD Web, or in other ways feel that “the COPD Web is for me”?<br>How do you perceive the COPD Web is to navigate/navigate to on the Internet?<br>How have you perceived the [films/information/how information is presented/the amount of information]?<br>How have/has [films/step registration/login/the COPD Web in general] worked/functioned for you?
Accompanying parts/”Triggers”	***Introduction***<br>How did you perceive the introduction of the COPD Web?<br>Did you look at the COPD Web together? (What did you look at/use?)<br>Is there something you feel could be done differently (related to the introduction)?
	Pedometer<br>What have you thought about getting a pedometer?<br>What is your experience with having the pedometer?<br>Do you feel that the pedometer has rendered you more physically active?
	e-mail newsletter<br>Have you gotten any e-mail newsletters from the COPD Web?<br>How do you perceive getting e-mail newsletters in this way?
Potential effects	If the COPD Web contributed anything to your life, what would it be?<br>Did you already know about these things (that the COPD Web informs about), or did you happen onto something new?
Future use	Is there anything on the COPD Web you feel could be enhanced/developed?<br>What are your thoughts around continuing using the COPD Web in the future?
Usual finishing question	Is there anything else you have thought about/would like to say about the COPD Web that I have not asked about?
